# Comparative genomics of fungal allergens and epitopes shows widespread distribution of closely related allergen and epitope orthologues

**DOI:** 10.1186/1471-2164-7-251

**Published:** 2006-10-09

**Authors:** Paul Bowyer, Marcin Fraczek, David W Denning

**Affiliations:** 1 Faculty of Medicine and Human Sciences, University of Manchester, Wythenshawe Hospital, Manchester, M23 9LT, UK

## Abstract

**Background:**

Allergy is a common debilitating and occasionally life threatening condition. The fungal kingdom contains a number of species that produce a wide range of well defined protein allergens although the vast majority of fungal species have unknown allergenic potential. The recent genome sequencing of a variety of fungi provides the opportunity to assess the occurrence of allergen orthologues across the fungal kingdom. Here we use comparative genomics to survey the occurrence of allergen orthologues in fungi.

**Results:**

A database of 82 allergen sequences was compiled and used to search 22 fungal genomes. Additionally we were able to model allergen structure for representative members of several highly homologous allergen orthologue classes. We found that some allergen orthologue classes that had predicted structural congruence to allergens and allergen epitopes were ubiquitous in all fungi. Other allergen orthologues classes were less well conserved and may not possess conserved allergen epitope orthologues in all fungi. A final group of allergen orthologues, including the major allergens Asp f 1 and Alt a 1, appear to be present in only a limited number of species.

**Conclusion:**

These results imply that most fungi may possess proteins that have potential to be allergens or to cross react with allergens. This, together with the observation that important allergens such as Asp f 1 are limited to genera or species, has significant implications for understating fungal sensitization, and interpreting diagnosis and management of fungal allergy.

## Background

Allergy is a common ailment seen with increasing frequency in the developing world [1-4]. Although allergies are known to be caused by an enhanced type 2 immune response the defining characteristics of the causative agents of allergenicity are not well understood. Many proteins responsible for allergic reactions have been described. Despite intensive efforts to determine what distinguishes these proteins from the other non-allergens in the same proteome, little is understood about the structural basis for allergenicity. We also do not understand why some organisms are often associated with allergy and other closely related organisms are never or only rarely observed to cause allergy. One explanation is that species not associated with allergy do not possess genes encoding allergen proteins. Alternatively allergen orthologues may be common in all organisms and allergenicity could be determined by rigorous sequence or epitope requirement, the context or timing of presentation of proteins to the immune system, or by third-party factors expressed by a colonising organism or present in the environment. However too few species have been studied in this way and it is likely that many undiscovered fungal allergens in fact exist.

A crucial first step in evaluating allergen distribution is to determine whether allergen orthologues are present in ostensibly non-allergen producing species and to what extent. Homology to existing allergen sequences is a useful, though imperfect, tool for prediction of whether an unknown protein may either be allergenic or cross reactive at either T cell, B cell or mast cell level. However homology to allergens can be determined for all proteins in a given proteome. Additionally proteins with high levels of homology to proteins with known structure can be readily modelled to allow examination of putative epitope structures or protein fold.

**Figure 1:**
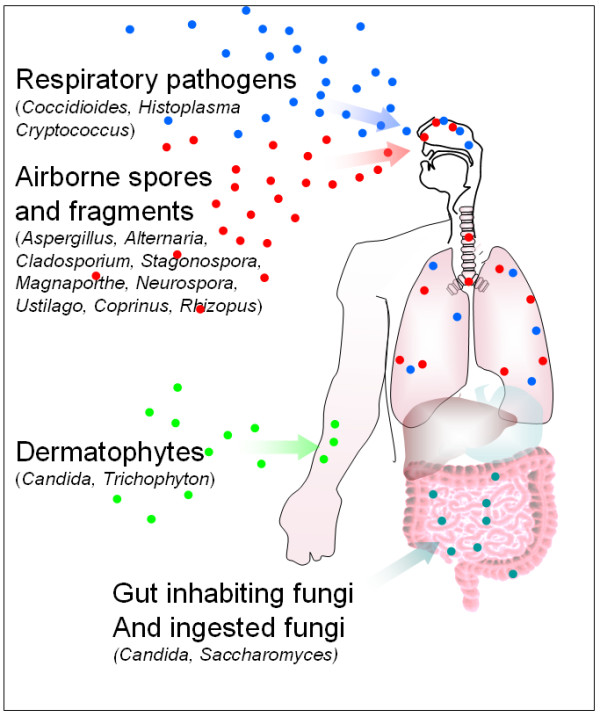
Sources of exposure to fungi and fungal allergens.

Fungi are an ideal group of organisms in which to investigate allergen orthologue distribution as they are a diverse kingdom comprising over an estimated million species, are common both in the environment and as epiphytes, pathogens, gut inhabitants and endophytes of man (Figure 1). Respiratory exposure to a wide range of fungal spores and fragments is almost constant [5-7]. According to allergen databases 189 fungal species are thought to produce allergens (Bowyer, unpublished observations). Several studies have linked exposure to high levels of fungal spores with episodes of asthma, some life-threatening [8]. Indeed we recently described substantial fungal loads in bed pillows [9] which implies frequent exposure to high levels of fungal spores or hyphal fragments.

Filamentous fungi contain an average of 10,000 genes whereas the less complex yeast-like fungi may contain only 6,000. A recent survey of fungal allergens (Bowyer unpublished observations) shows that the best studied fungi may have up to 20 known well characterised allergens (in the case of *Aspergillus fumigatus*, *Cladosporium herbarum *and *Alternaria alternata*), between 27 and 60 other less well characterised IgE binding proteins as determined by IgE binding to phage displayed allergen libraries [10-12] and another 20 proteins predicted to be allergen orthologues by virtue of close homology to allergens known in other species (this publication). Thus an estimated 0.5 – 1% of proteins in a given fungal proteome may be allergens. The known fungal allergens appear to occur as functional groups such as serine proteases, heat shock proteins or thioredoxins or orthologues of proteins such as Mn superoxide dismutase or enolase. The best studied fungi appear to possess allergen proteins from most of these classes whereas the less well studied fungi are only known to possess allergens from a small number of these groups. This suggests that more allergens are discovered as an organism is studied more which implies that fungi may all possess a core set of allergen classes. A further group of allergens containing the major allergens Asp f 1, Alt a 1 and Cla h 1 have only been found in fungi from particular genera. Many diverse members of the kingdom have publicly available genome sequences and recently the first genome sequence of an allergenic organism, the fungus *Aspergillus fumigatus*, was determined [13]. *A. fumigatus*, *C. herbarum *(Ch) and *A. alternata *(Aa) have been shown to be capable of being causative agents of allergy in murine models [14-16]. Fungi such as *Histoplasma*, *Cryptococcus *and *Coccidioides *are well described primary pulmonary pathogens that have a long term intimate relationship with their host but have never been observed to produce any allergic reaction during the course of human infection [17] although we note that *C. neoformans *has profound immunomodulatory effects in immune competent rodents [18]. Other genera are common skin or gut inhabitants such as *Candida *or *Trichophyton *and have been shown to produce allergens and may also be the causative agents of allergic reactions [19]. A final group of fungi including *A. fumigatus *(Af) and *Candida *spp. are opportunistic pathogens capable of causing invasive disease in immunocompromised hosts [14]. However the vast majority of fungi have never been described to contain allergens or be pathogenic.

Many potent allergenic proteins have been described in Af, and this organism is the first allergen-containing species to be sequenced [13,20] so we used this organism as the basis for comparative genome analysis. Reviews of allergen sensitization conducted in geographically diverse areas suggest the most common causes of fungal sensitization in populations are *Aspergillus*, *Alternaria *and *Cladosporium *spp. These genera together with *Penicillium *are frequently among the most common fungi encountered in surveys of airborne fungi in indoor and outdoor environments worldwide [21-26]. However many other fungal species are also commonly found in these studies with unidentified non-sporulating fungi being highly represented. A recent survey of spore levels in the UK suggests that the combined level of *Aspergillus*, *Penicillium*, *Alternari*a and *Cladosporium *spores forms approximately 15% of the total airborne fungal matter, much of the rest of which is accounted for by ascospores and basidiomycetes [25]. Another recent study shows the high levels of Af, *Aureobasidium pullulans *and *Rhodotorula mucilaginosa *in bedding [9].

The genome sequences of *A. fumigatus *[13] and two other closely related species of *Aspergillus *(*A. oryzae *(Ao) and *A. nidulans *(An)) [27,28] have been recently determined allowing analysis of allergen orthologues across this genus and ~20 other species of fungus. The comparison between the well known allergen producing fungus Af and Ao or An is particularly interesting. An is an environmentally common fungus not previously observed to produce allergens, although Asp n 2 which is orthologous to Asp f 2 may be a possible candidate. Ao has been used intensively in fermentation of soy sauce for over 2 thousand years with few reported cases of allergy although 'soy sauce workers' lung' is now an accepted form of extrinsic allergic alveolitis. The genomic analysis was also extended to compare the genomes of the other sequenced fungi with varying pathogenic lifestyles to demonstrate whether allergen orthologues are truly ubiquitous in the kingdom or whether they are restricted to the species or genera that are known to produce allergens. Representative allergen orthologues were structurally modelled and orthologous epitope structures determined to discover whether they could possibly function as cross reactive proteins or even allergens. The results of this analysis show that many groups of allergen orthologues are ubiquitous in fungi.

## Results

### Allergen orthologue distribution in the genus *Aspergillus*

**Figure 2:**
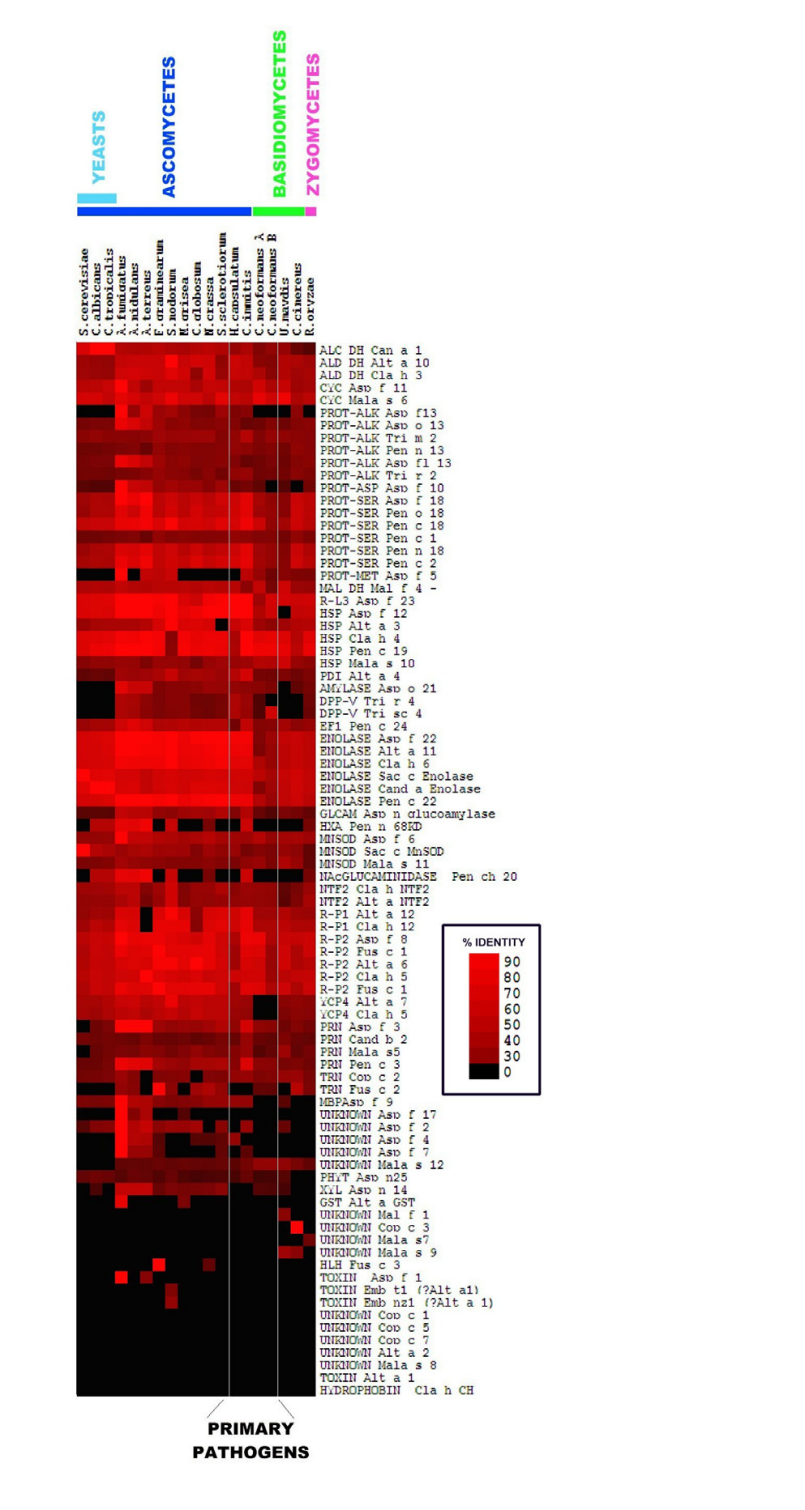
**A Heat map showing homologies of fungal allergen genes to fungal genomes**. The heat map was constructed from an MS excel file using Cluster and Treeview. Identities are coded by increasing colour saturation with bright red denoting the highest degree of identity.

As expected, Af allergen sequences were present in the Af genome sequence and in the predicted Af proteome (Figure 2). All allergen database sequences showed 100% identity to the genome sequence and predicted peptides. All allergen homologues from Ao and An are >60% identical with f 3, f 5, f 7, f 8, f 11, f 12, f 18, f 22 and f 23 all having >70% identity. This suggests that An and Ao could produce many proteins cross-reactive to those of Af. Ao has been reported to produce as many as 13 IgE reactive proteins and an allergen Asp o lipase. [29] Only two Af allergens, Asp f 1 and Asp f 5 did not have homologues in both An and Ao although Asp f 5 did have a close homologue in Ao. The significance of this observation awaits experimental elucidation however a naïve hypothesis could be that Af is most commonly observed in allergy due to the presence of Asp f 1. Asp f 1 has no orthologs in the *Aspergillus *genomes analysed here but closely related proteins are known to occur in *A. giganteus *(83% identity) and *A. clavatus *(81% identity) [30]. We note the occurrence of a gene in *A. terreus *with 41% identity to Asp f 1 which may be a non-orthologous non-toxin RNAse. The reported existence of at least 13 IgE reactive proteins in Ao is consistent with our observation that Ao possesses 16 orthologues closely related to Af allergens.

### Allergen orthologues are present across the fungal kingdom

**Figure 3:**
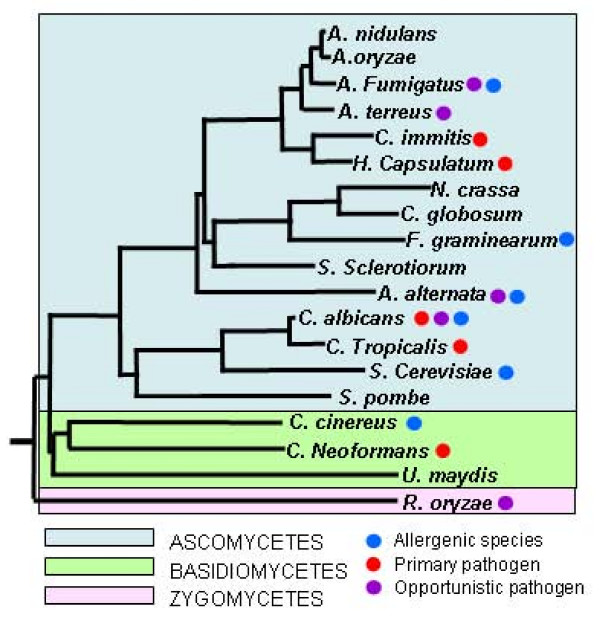
**Phylogeny of fungi included in this study**. Tree constructed from 18S ribosomal sequences available from Genbank. using CLUSTALW and phylip software.

In order to determine whether allergen orthologues with high levels of homology occur throughout the fungal kingdom the allergen database was used to search the genomes of 12 further ascomycetes: *Neurospora crassa*, *Fusarium graminearum*, *Magnaporthe grisea*; *A. terreus*, *C. albicans, C. tropicalis, Chaetomium globosum, Saccharomyces cerevisiae, Sclerotinia sclerotiorum, Stagonospora nodorum, H. capsulatum*, and *C. immitis*; 4 basidiomycetes: *Ustilago maydis, C. neoformans *serotype A, *C. neoformans *serotype B and *Coprinus cinereus *and the zygomycete, *Rhizopus oryzae*. The results are represented by the heat map in Figure 2. Evolutionary relationships of the fungi used including non-sequenced major allergens are shown in Figure 3. This shows that fungi share a highly conserved set of allergen orthologues. The conserved group of allergens include enolase, heat shock proteins, cyclophilins, proteases, redoxins, and disulphide isomerases. In general homologies correlate with evolutionary relatedness. The other ascomycetes, including the opportunistic pathogens, *A. terreus*, *A. fumigatus *and *Candida *appear to retain a full complement of allergen-like proteins, apart from a few key allergens including Alt a 1, Alt a 2, and Asp f 1 which appear to be highly specific to particular allergenic species, genera or families.

### Computer modelling of allergen structures

**Figure 4:**
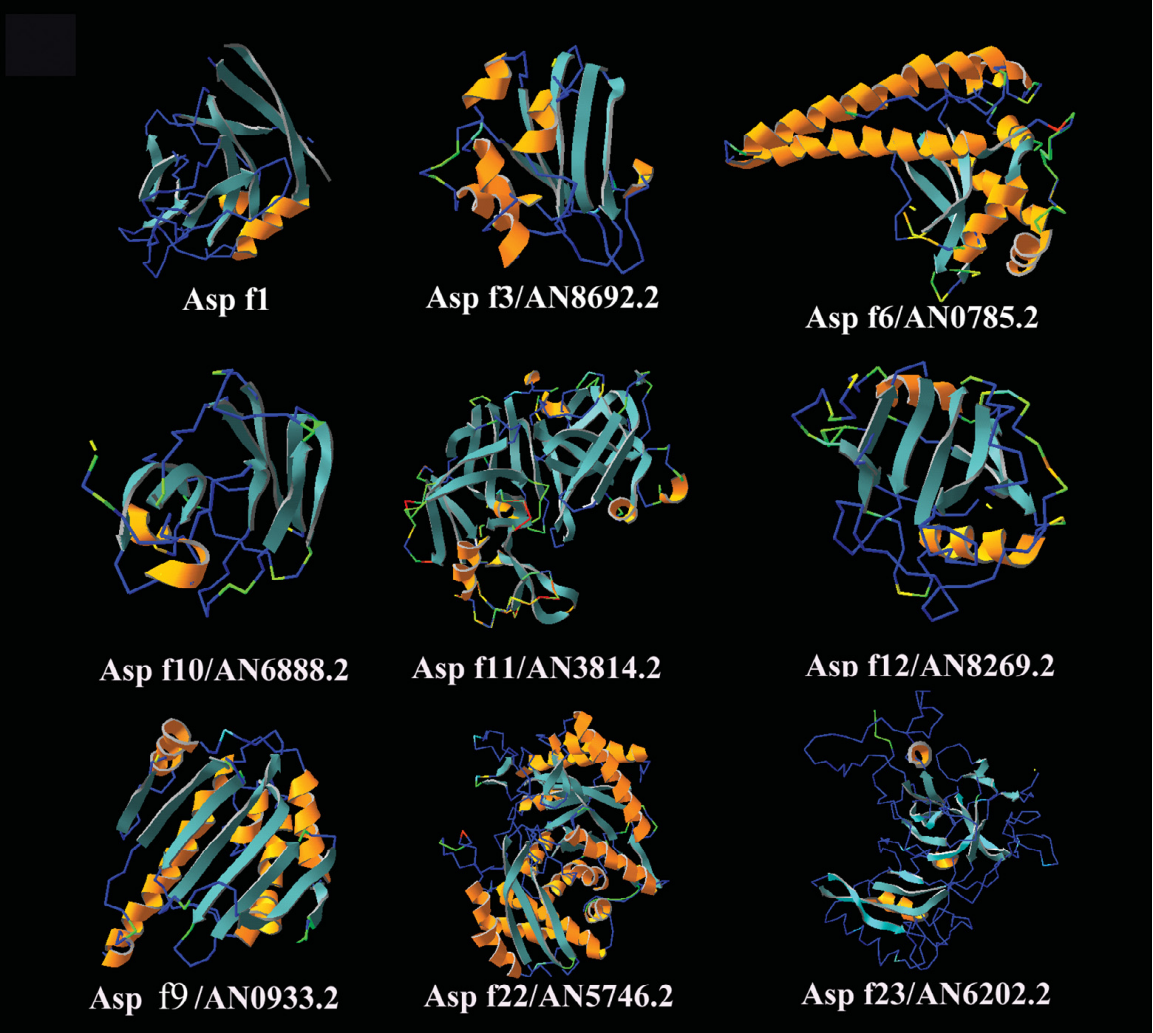
**Structural models of *Aspergillus *allergens**. Panels show *A. fumigatus *and *A. nidulans *models superimposed. Backbone structures are near identical and indistinguishable at this scale. Conserved residues in the backbone are shown in blue, divergent amino acids are shown in green and yellow to indicate structurally divergent side chains.

Clearly the presence of genes with homology to known allergens does not by itself prove that the proteins produced are cross-reactive or even allergenic. A minimum requirement for consideration as possible allergens or cross reactive proteins is that the orthologues share at least some structural features with known allergen proteins. Surveys of allergen cross-reactivity suggest that cross-reaction commonly occurs in proteins having >50% identity levels [31-35]. Additionally this level of identity is known to give rise to proteins with near identical 3-dimensional structures [36,37]. Thus it is useful to determine whether the orthologues share structural features with known allergens. Another consideration is that candidates for allergenicity should retain both epitope sequence and structure. We note that presence of such epitope orthologues is no guarantee that IgE will bind at these sites in a cross reactive manner as most of the epitopes determined in fungi are linear whereas B-cell epitopes are usually considered to be structurally distributed. However consideration should be given to the presence of such epitope orthologue regions until such time as accurate distributed epitope regions are delineated on fungal allergens by IgE co – crystallisation studies. In order to demonstrate that the homologous proteins may contain epitope orthologues in 3-dimensional configurations that share the structural organisation of known allergen epitopes, we constructed *in silico *models for Af allergen molecules based on highly homologous proteins (>60% identity) with known structures (PDB files for enolases: 4ENL_, serine proteases: 1IC6A, ribotoxin:1AQZ, MnSOD: 1KKC). We then modelled structures for orthologues from sequenced species not known to produce allergens and for other known allergen proteins from species that do not have known genome sequence such as Ch, Aa and *Penicillium *spp. Using these structures, we delineated the epitope orthologues regions for protein classes that have both known epitopes and a structural model in order to investigate whether the potential epitope orthologues from non-allergen species retained both amino-acid sequence identity and 3D structural congruence with those in allergenic species. Only two experimentally determined structures of Af allergens – Asp f 6 and Asp f 8 – are published [38,39]. Af structural models were constructed based on the closest experimentally determined structure. To show that overall protein structure was conserved between known allergens and non-allergens from different species we initially compared backbone structures of Af allergens and An allergen orthologues. An is abundant in nature [20-26] but no allergens have been described in this organism. Protein models were constructed based on primary structural models except in the case of homologues of Asp f 6 and f 8 which were modelled on the published Asp f 6 and f 8 structures. Figure 4 shows a comparison of predicted structures between An orthologues and Af allergens. A number of Af and An structures could be modelled with high confidence including Asp f 1, Asp f 3, Asp f 6, Asp f 10, Asp f 11 and Asp f 12 and their homologues. Af and An structures are shown superimposed in Figure 4. The predicted backbone structure of *An *allergen orthologues cannot be distinguished from *Af *allergens for all models.

**Figure 5:**
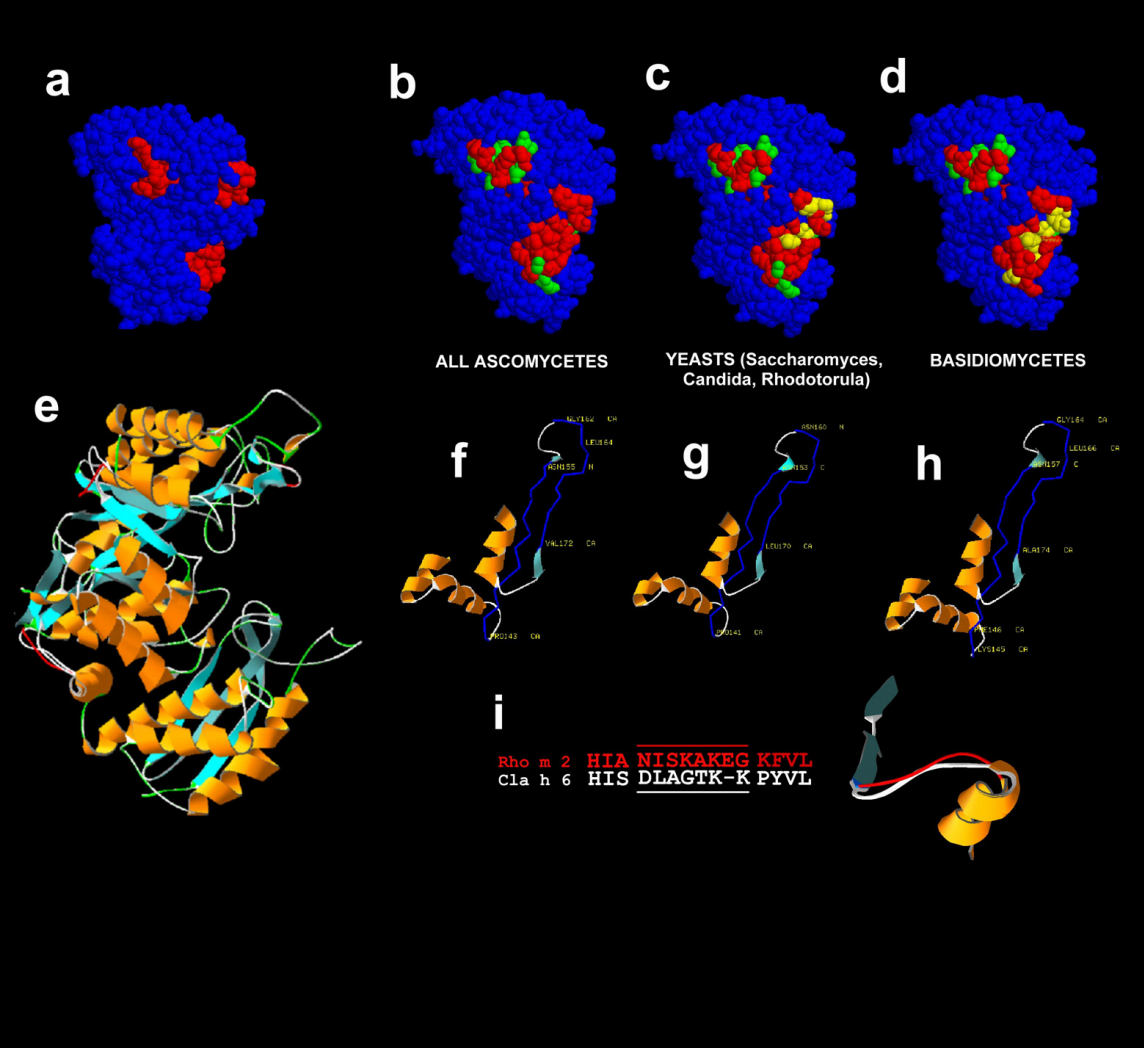
**Structural models showing conservation of allergen and epitope structure between enolase allergens and allergen homologues**. Conserved non-epitope structure is shown in blue. Epitope structure that is identical in all allergens studied and also identical in allergen-like proteins is shown in red. Epitope amino acids not conserved between experimentally determined cross-reacting allergen epitopes are shown in green and amino acids differing from epitope in allergen-like proteins are shown in yellow. A: View of enolase Cla h 6 predicted structure as a space fill model showing conserved epitope present in all fungi. B: Reverse view of Cla h 6 showing epitopes conserved in ascomycetes C: Reverse view of Cla h 6 showing epitopes conserved in yeasts, *C. albicans, C. tropicalis, S. cerevisiae *and *R, mucilaginosa*. D: Reverse view of Cla h 6 showing epitopes conserved in basidiomycetes *C. cinereus C. neoformans *and *U. maydis *E: Representative enolase structures consisting of Cla h 6, Sac c enolase and *C. cinereus *enolases superimposed. F: Enolase epitope structure for ascomycetes. The exposed epitope structure is displayed as ribbon and the buried portion of the epitope is shown as a blue CA trace. G: Enolase epitope structure for yeasts. The exposed epitope structure is displayed as ribbon and the buried portion of the epitope is shown as a blue CA trace. H: Enolase epitope structure for basidiomycetes. The exposed epitope structure is displayed as ribbon and the buried portion of the epitope is shown as a blue CA trace. I: Structure and amino acid sequence of the divergent loop in yeast and basidiomycete epitopes (circled in C, D and E)

**Figure 6:**
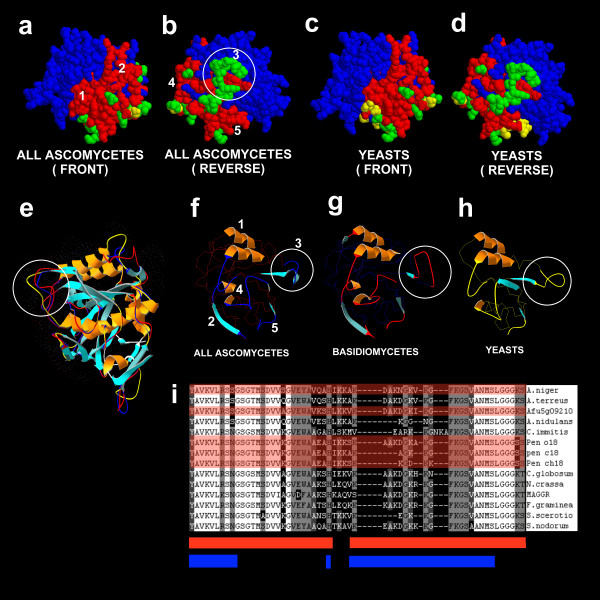
**Structural models showing conservation of allergen and epitope structure between serine protease allergens and allergen homologues**. A: View of protease Pen ch 18 predicted structure as a space fill model showing conserved epitope present in all fungi. B: Reverse view of Pen ch18 showing epitopes conserved in ascomycetes C: View of Pen ch 18 showing epitopes conserved in yeasts, *C. albicans, C. tropicalis, S. cerevisiae *and *R, mucilaginosa*. D: Reverse view of Pen ch 18 showing epitopes conserved in yeasts. E: Representative protease structures consisting of Pen ch 18, *C. albicans *homologue and *C. cinereus *models superimposed. F: Protease epitope structure for ascomycetes. The exposed epitope structure is displayed as ribbon and the buried portion of the epitope is shown as a blue CA trace. G: Protease epitope structure for basidiomycetes. The exposed epitope structure is displayed as ribbon and the buried portion of the epitope is shown as a red CA trace. H: Protease epitope structure for yeasts. The exposed epitope structure is displayed as ribbon and the buried portion of the epitope is shown as a yellow CA trace. I: Amino acid sequence pile up of the divergent loop in ascomycete epitopes (circled in B, E, F, G and H). Epitope regions are shown as a red bar underneath the pileup and surface exposed residues are shown by the blue bar. Allergen sequences are indicated by the red overlay.

**Figure 7:**
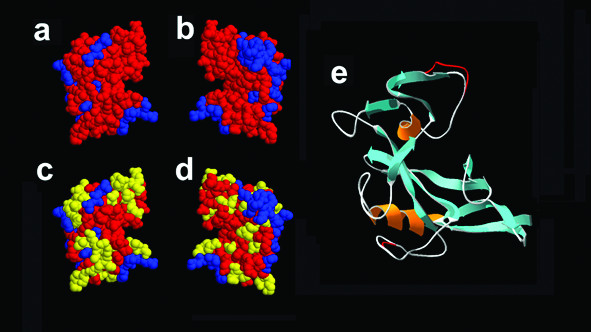
**Structure and epitope identity of the Asp f 1 homologue from *A.terreus***. Epitope and structure were mapped for the *A. terreus *Asp f 1 homologue as previously described. A. Asp f 1spacefill structure showing epitope regions in red. B: Reverse Asp f 1spacefill structure showing epitope regions in red. C: *A. terreus *Asp f 1 homologue spacefill structure showing epitope regions in red with divergent amino-acids in yellow. D: Reverse *A. terreus *Asp f 1 homologue spacefill structure showing epitope regions in red with divergent amino-acids in yellow E: Predicted ribbon structures of Asp f 1 and *A. terreus *Asp f 1 homologue superimposed. Two divergent *A. terreus *loop structures are shown in red.

Here we have chosen well characterised epitopes from known allergens to illustrate how epitope structure might be conserved across the families of fungal allergen orthologues. In our examples we have chosen proteins that can be modelled from highly homologous templates and which have mapped epitopes (enolases, subtilisin type proteases and ribotoxin), these are shown in figures 5, 6 and 7 and described in the following 8 paragraphs.

Enolase epitopes have been determined in *Candida *and *C. herbarum*. [40,41]. The well characterised Cladosporium (Cla h 5) enolase epitope has been shown to be cross reactive with Alt a 5, Cand a enolase and Asp f 22 and was thus chosen for this analysis. Known fungal enolase allergens include Asp f 22, Pen c 22, Cand a Enolase, Rho m 1, and Sac c Enolase. The peptide containing the epitope consists of a region of the protein with buried structure and three surface regions. Here we compare only surface residues as they are likely to be important for IgE binding although buried residues may also play a structural role in determining the surface structure. However buried structure was also modelled and can be seen to be highly conserved in all models.

Subtilisin protease epitopes have been determined in Pen c 18 [42,43]. The subtilisin-like protease allergens and orthologues typified by Asp f 18 display highly conserved epitope and epitope orthologues structures (Figure 7). In this case we chose to map the epitope sequences reported by Yu and co-authors [42].

The ribotoxin allergen Asp f 1 appears to be highly conserved across a small clade of Aspergilli including *A. fumigatus *and *A. clavatus *but not present in other members of the genus. We note the presence of a low homology orthologues of Asp f 1 in the *A. terreus *genome – an organism previously reported to have no cross reactivity to Asp f 1 [29]. Hence we examined the predicted structure and epitopes of Asp f 1 and its orthologues to determine whether Asp f 1 epitope orthologues were conserved and whether the orthologues from *A. terreus *would be predicted to contain credible epitope orthologues using our approach [44,45].

Spacefill models were used to show position and relatedness of amino-acids making up the experimentally determined epitopes. When epitope and epitope orthologue regions were examined we noted that epitopes from allergens with known cross reactivity often contained differences and we reason that these residues in the epitope are not essential for IgE binding or that certain types of substitution may not affect epitope activity. One possible problem with this assumption is that cross reactivity is always determined using polyclonal IgE sera as monoclonal IgE is not generally available for such studies. This raises the possibility that the differences noted between cross reactive epitopes may reflect binding by different IgEs. This problem could be resolved by obtaining monoclonal IgE against the epitopes using phage display libraries constructed from individuals. Comparison of epitopes and epitope orthologues also revealed residues in the epitope orthologues that were clearly different to conserved epitope sequences.

Amino acids that are completely conserved in epitopes are marked in red. Epitope amino-acids known to vary among experimentally determined cross reactive epitopes and therefore probably not involved directly in IgE binding are shown in green. Amino acids that are different to the conserved epitope structure are shown in yellow. It appears that some epitopes or epitope orthologues are highly conserved or identical in both linear amino acid sequence and conformational structure across the fungal kingdom. For example enolase epitope region 1 (Figure 5A) is 100% identical in all fungal species tested and appears to retain the same conformation. Enolase epitope region 2 (Figure 5B,C,D) contains regions that are invariant and regions that vary amongst cross reactive allergens (marked green) as well as residues that are different to the conserved epitope sequence (yellow) although these divergent residues are not fully exposed on the surface of the protein. Epitope region 3 (Figure 5B,C,D) is invariant amongst the ascomycete fungi but varies structurally in yeasts, Zygomycetes and Basidiomycetes by containing a divergent loop structure (Figure 5E–I). The pileup showing amino acids conserved and diverging in the epitope orthologues on which the spacefill colours are based is shown in figure 5J.

Epitopes 1 and 2 (Figure 6A and 6C) are highly conserved in all fungi whereas epitope regions 4 and 5 (Figure 6B and 6D) are conserved in ascomycetes but show some divergence in yeasts (Figure 6C and 6D) and basidiomycetes [not shown]. Additionally the spacefill model is unable to show structural divergence in epitope 3 (circled in Figure 6A) which is shown circled in the ribbon structures in Figure 6E–H. This region is highly variable in all fungi (Figure 6I) and shows considerable variance amongst allergens with known cross-reactivity. Other non-epitope surface loop regions in the structures (Figure 6E) are also different when compared between yeasts (yellow backbone in Figure 6E), ascomycetes (blue backbone) and basidiomycetes (red backbone). Thus for the Asp f 18 class of serine proteases the protein is highly conserved for filamentous ascomycetes and could be cross-reactive for all higher fungi.

The only species in our analysis with a potential Asp f 1 orthologue was *A. terreus *which has been specifically shown not to serologically cross – react with Asp f 1 [29]. The predicted amino acid identity with Af is 41%. When we compared the Asp f 1 structure and epitopes with the homologue detected in *A. terreus *we found that the predicted structure was similar but that the epitope orthologues sequence was not conserved (Figure 7A–E). In this case, the homology between model and experimentally determined structure was low (41%) and we have limited confidence in the structure shown despite it being the best possible alignment to Asp f 1 for epitope orthologue conservation. The considerable differences in surface composition between the *A. terreus *and Af proteins are evident and suggest a likely explanation for lack of immunoreactivity of *A. terreus *extracts to Asp f 1 antibody.

In general, variable regions in the allergen orthologues are also variable in cross – reactive allergens while conserved regions are conserved in all proteins. Clearly not all homologies detected in this study will result in conserved epitopes or structures and we are unable to predict those that will be important from the two examples presented in figures 5 and 6 however we would suggest that the majority of proteins with >50% identity have the potential to be cross-reactive or allergenic representing a vast number of proteins in the fungal kingdom.

## Discussion

Comparative genomics was used to compare allergens between different closely related species and to study the presence of potential cross-reacting proteins or allergen orthologues in fungi generally. We found that not just the genus *Aspergillus*, but all fungi possess a core set of allergen orthologues. Where we were able to determine both orthologue structure and epitope orthologue structure we found congruence at both the gross structural and the epitope orthologue structure levels throughout fungi although for some epitopes the epitope orthologue identity only occurred across the ascomycete or basidiomycete groups. In these cases however we note that previously published experimental evidence shows that proteins previously described as allergens and containing these divergent allergen structures are serologically cross reactive with each other [20,39-43]. We also note several reports of cross reactivity to extracts from various different fungal species that may broadly support the suggestion that many species of fungi contain cross reacting classes of protein [46-50]. Finally there are several reports of cross reactivity between structurally characterized proteins from different fungal species or between fungal allergens and human proteins [51-59]. Thus there is considerable evidence indirectly supporting the hypothesis that all fungi may contain allergen orthologues that may cross react. Clearly the nature of published literature means that reports of lack of cross reactivity between species extracts or proteins are rare and we therefore urge caution in interpretation of these results.

Although cross reactivity between fungal proteins with homology levels between 40 and 70% is well reported there are other examples in the literature such as the birch tree allergen Bet v 1 and its hypoallergenic isoform Bet v1 l which show that single amino acid changes or differences at surface residues distant from the epitope may significantly reduce reactivity [60-62]. Epitope – IgE interaction can also be affected by underlying protein structure thus it is certainly possible that the epitope orthologues shown here may not be functional. It has been shown that 6 of 9 Bet v 1 isoforms with >98% identity were moderate – high IgE binding when tested against the same serum pool with the remaining three showing little or no IgE binding [62]. Thus relatively small changes in protein sequence may affect cross reaction. This data is subject to some interpretation as recent findings show that birch may express a number of paralogues of Bet v 1 and it is unclear which of the isoforms tested were orthologous isoforms or simply different paralogues [63]. It is probable that in this case the paralogues for this defense protein have subtly different function and structure which may affect their reactivity with IgE [66,67]. It is also possible that single amino acid changes can affect overall structure of a protein which would drastically affect IgE binding [68,69].

In contrast to such experiments showing that relatively minor sequence changes may affect IgE binding other experiments have shown that four of 6 Bet v 1 orthologous allergens and proteins with >60% identity in related species are cross reactive. Cross reactivity has been shown between Bet v 1 and the celeriac allergen Api g 1. Studies show that Api g 1 specific T cells generated with Apig1 cross-react with Bet v 1 and that cellular responses to Betv1 may be stronger than to Apig1. Betv1_109–126 _was identified as the most important Tcell epitope for cross-reactivity with Apig1 [64,65]. This epitope shares only 72% amino acid sequence similarity with the major Tcell-activating region of Apig1_109–126._

Thus it appears that some important single residue polymorphisms as well as large distributed differences in homology may have variable effects on IgE binding. The overwhelming majority of allergens and proteins that show cross reactivity are closely related at the amino acid level and thus we propose that our level of >50% identity to a known allergen at the amino-acid level is usually necessary for a protein to qualify for consideration as an allergen. Retention of the same fold structure and epitope orthologue conformation as the allergen is also required. It is important to note the probable existence of protein surface epitopes that have not yet been described so that presence or absence of epitope orthology based on known epitopes may not be a sufficient qualification. Having graduated from these criteria the protein may still not function either as an allergen or an IgE cross reactive protein because the differences in protein sequence may confer subtle changes in protein structure that render the protein either unable to initiate allergenicity or to bind IgE effectively. Surveys of cross reactive proteins and allergens related at the sequence level show that such proteins with >50% identity usually cross react. In fungi we note that all proteins with >50% identity to known allergens that have been studied are found to be either cross-reactive or allergenic. We would thus argue that the highly similar epitope orthologues presented here that are embedded in proteins that often have >70% identity to known allergens may often represent IgE binding sequences. Clearly other structural features such as post-translational modifications may be relevant in determining whether a protein can be allergenic.

This cross species comparison predicts the presence of a number of allergen orthologues in Af. During the preparation of this manuscript, four new IUIS approved Af allergens were described (Asp f 27 – cyclophilin, Asp f 28–29 – thioredoxins, and Asp f GST) [70] that were found in our analysis as allergen orthologues on the basis of their homology to other fungal allergens. This finding supports the idea that the allergen orthologues found in this study may in fact have the potential to be allergens. The organisms in this analysis represent an allergenically random sample of fungi, as their selection for genome sequencing was for other reasons;- pathogenicity to plant or animal, status as a model genetic organism or as representatives of a particular morphology or growth habit [see Figure 3]. Therefore, we would suggest that they can be taken, with certain exceptions, to be representive of the majority of the fungal kingdom. Some epitope orthologue structures and sequences are completely conserved in all fungi. Therefore this analysis may indicate the presence of allergen orthologues in over a million species of fungi, many of which are in everyday contact with the skin, gut and respiratory mucosa.

The observation that most reported fungal allergens come from only three species may have several explanations. The most obvious is that these are the best studied and most commonly encountered fungi. Other possibilities are that these fungi have special characteristics that make them suitable for presentation of allergen proteins such as production of 3^rd ^party mycotoxins, ability to colonize the lungs or gut or high level expression of the allergen genes.

Af is well recognized a long-term saprophyte and colonizer of the respiratory tract (ABPA, aspergilloma and maxillary fungal sinusitis), *A. alternata *can be an opportunistic and superficial nail pathogen and *Cladosporium *and *Penicillium *spp. can occasionally be pathogens [72,73]. Thus the capability to survive in or on the host for enough time to synthesize sufficient allergenic protein at the right time and in the right place may be a necessary for the expressed allergen proteins to produce allergenicity. In contrast, primary pathogenic fungi such as *C. immitis, H. capsulatum *and *C. neoformans *that attack and dwell in the lung for long periods have never been observed to cause allergic reactions in man. Like other fungi, they possess a large complement of allergen orthologues proteins with structurally conserved epitopes. It is possible that these primary pathogenic fungi do not present the allergen orthologues proteins at the right time in vivo or insufficient quantity to trigger allergy or that such highly adapted organisms are capable of interfering with the IgE response which in its proper state is a potent weapon against pathogen attack.

Many major allergenic species possess allergens that are unique to that species or its close relatives. Examples of this are Asp f 1, Alt a 1 and Cla h 1. These proteins may play an important role in making a species allergenic. For example Asp f 1 is a ribotoxin that may prime the atopic host for allergic reaction by damaging epithelial tissue. However our analysis is limited to homologues of known allergens and it is possible that all fungi contain such species limited toxins or allergens. Recent analysis of fungal proteomes indicates that there are many proteins that are unique to each genus so far studied.

It is possible that the particular allergens themselves are intrinsically more allergenic in these species compared with other fungi. Given the basic similarity of the proteins at the structural level, many published instances of cross-reactivity, pragmatically determined level of identity giving cross-reactivity and the conservation of epitope structures across the fungal kingdom it seems probable that at least some of the allergen orthologues will be serologically cross-reactive or allergenic if presented to the immune system appropriately. It is known that the protein 3D structure underlying or surrounding an epitope is important in IgE binding but these regions are also highly conserved. Cloning of genes encoding allergens has revealed that most, if not all, allergens cloned from different isolates of a single species are heterogenous with variation in up to 25% of the amino acids in an allergen orthologues with known allergen activity [71]. The particular feature of these classes of proteins that makes them allergens rather than the other 99% of cellular proteins may well arise from complex distributed protein features.

The presence of multiple close allergen homologues across the fungal kingdom carrying epitope orthologues that are structurally indistinguishable from IgE binding epitopes may raise a considerable problem in diagnosis and classification of fungal allergy. Even allowing for the fact that many of these allergen orthologues may be neither allergenic nor cross-reactive the sheer size of the fungal kingdom means that the remaining number of fungi that would contain allergens or cross reactive proteins is likely to be huge. In such a case the common practice of screening for allergens using reactivity to serum IgE may be fraught with difficulties and determining the primary agent that is causing an allergy may more difficult than is currently realised. We note however that the various important allergen containing species all possess subsets of major allergens such as Asp f 1, Alt a 1 and Cla h 1 that are specific to species or at least genus. These allergens would provide a species or genus specific diagnostic and are already used in some cases. The efficacy of using culture filtrate from fungi in skin prick testing or IgE binding assays would then depend on the proportion of species or genus specific allergens present in the crude mixture – high proportions of species specific allergens giving the most precise result. It might be useful to perform skin prick tests with mixtures of species specific allergens from the same fungus in order to achieve a more precise result.

## Conclusion

In conclusion this analysis suggests that fungal allergen orthologues are abundant in nature and that many of them occur in the majority of fungal species. It seems likely that many of these allergen orthologues are potential allergens or at least capable of cross reactivity at some level of the immune response. This finding suggests that the frequency of exposure, persistence, context of presentation or provenance of the allergen protein are important in determining how frequently a particular species is encountered as a cause of allergy.

## Methods

### Databases and analysis

A database of 82 non-redundant fungal allergen sequences was compiled from 102 fungal allergen protein sequences from databases at Allergome [70], SDAP, Allermatch [75] and SwissProt [76]. The non redundant list contained the following allergen sequences: Alt a 1, Alt a 3, Alt a 4, Alt a 5, Alt a 6, Alt a 7, Alt a 8, Alt a 10, Alt a 12, Cla h 2, Cla h 5, Cla h 6, Cla h 7, Cla h 8, Cla h 9, Cla h 10, Cla h 12, Asp fl 13, Asp f 1, Asp f 2, Asp f 3, Asp f 4, Asp f 5, Asp f 6, Asp f 7, Asp f 8, Asp f 9, Asp f 10, Asp f 11, Asp f 12, Asp f 13, Asp f 17, Asp f 18, Asp f 22w, Asp f 23, Asp n 14, Asp n 18, Asp n 25,, Asp o 13, Asp o 21, Pen b 13, Pen b 26, Pen ch 13, Pen ch 18, Pen ch 20, Pen c 3, Pen c 13, Pen c 19, Pen c 22w, Pen c 24, Pen o 18, Fus c 1, Fus c 2, Tri r 2, Tri r 4, Tri t 1, Tri t 4, Cand a 1, Cand a 3, Cand b 2, Psi c 1, Psi c 2, Cop c 1, Cop c 2, Cop c 3, Cop c 5, Cop c 7, Rho m 1, Rho m 2, Mala f 2, Mala f 3, Mala f 4, Mala s 1, Mala s 5, Mala s 6, Mala s 7, Mala s 8, Mala s 9, Mala s 10, Mala s 11, Mala s 13, Epi p 1. The database was compiled as a FASTA file and used to search locally stored and formatted genome peptide database files using NCBI formatdb and NCBI standalone BLAST2.0 [77]. BLASTP, TBLASTN and BLASTX [78] were used to search nucleotide and protein sequence databases. For cases were no match was found te results were checked manually to ensure tat sequencing errors were not responsible. BLAST data was parsed using BLASTEXTRACTER (MolGen Software, Department of Genetics, Groningen Biomolecular Sciences and Biotechnology Institute GBB), the Netherlands] and transferred to MS Excel for further analysis. Heatmap and cluster analysis was performed using CLUSTER and TREEVIEW programmes [79]. Genome sequence for comparison was obtained from TIGR [13,27,28] (*A. fumigatus*, *A. nidulans *and *A. oryzae*) with annotations provided by CADRE [80], The Broad Institute (*A. terreus, Candida albicans, C. tropicalis, Chaemotium globosum, Fusarium graminearum, Histoplasma capsulatum, Sclerotinia sclerotiorum, Neurospora crassa, Magnaporthe grisea, Ustilago maydis, Stagonospora nodorum, Rhizopus oryzae, Cryptococcus neoformans *serotype A and B, *Coccidioides immitis *and *Coprinus cinereus*) [81] and The Yeast Genome Database [82].

### Construction of structural models

Structural models were made using automated and manual modelling routines in an iterative manner using DeepView v3.7(SP5) [83,84] for alignment and realignment of sequences and energy minimization of models and Swiss Model [84] software and servers for threading of models. Gap regions were adjusted manually in DeepView. Ramachandran plots were used to check sequences during and after modelling.

Epitope sequences were obtained from previously published literature and manually mapped onto spacefill models of allergens using CLUSTALX v1.83 [85] to align sequences, Genedoc v2.6.002 [86] to visualise the resulting pileups and Rasmol v2.7.2.1.1 [87] to colour structures.

## Authors' contributions

PB carried out database analysis and setup, MF prepared heat map figures. PB and DWD contributed to design and conception of the analysis and wrote the text. All authors have read and approved this manuscript.
